# Association of the Physical Activity–Sitting Time Balance Index with Metabolic Syndrome and Depressive Symptoms in Korean Adults: KNHANES 2014–2024

**DOI:** 10.3390/healthcare14213714

**Published:** 2026-11-02

**Authors:** Youngyun Jin, Donghyun Kim

**Affiliations:** 1 Department of Sport Science, Sungkyunkwan University, Suwon 16419, Republic of Korea; player53@skku.edu; 2 Department of Sport and Health Science, Hanbat National University, Daejeon 34158, Republic of Korea; 3 Department of Big Data and Healthcare Convergence, Hanbat National University, Daejeon 34158, Republic of Korea

**Keywords:** physical activity, sedentary behavior, metabolic syndrome, depression, health surveys

## Abstract

**Highlights:**

**What are the main findings?**

- The physical activity–sitting time balance index was inversely associated with metabolic syndrome and depressive symptoms in 26,716 Korean adults, and both associations remained essentially unchanged after mutual adjustment.
- Among active adults, doubling physical activity and halving sitting time were equivalently associated with metabolic syndrome, whereas only sitting time was associated with depressive symptoms.

**What are the implications of the main findings?**

- A simple ratio of physical activity to sitting time may serve as a summary indicator of cardiometabolic health among physically active adults, pending prospective validation.
- For mental health outcomes and inactive populations, sitting time should be assessed separately because the ratio index discards this information.

**Abstract:**

**Background/Objectives:** The physical activity–sitting time balance index (PASTBI), the ratio of weekly physical activity to daily sitting time, has been linked to mortality. As a ratio, it implicitly assumes that proportional changes in the two behaviors carry equal and opposite weight. We tested this assumption while examining associations with metabolic syndrome and depressive symptoms. **Methods:** We analyzed 26,716 adults from the Korea National Health and Nutrition Examination Survey 2014–2024. Metabolic syndrome was defined by the revised National Cholesterol Education Program criteria and depressive symptoms by a Patient Health Questionnaire-9 score ≥ 10. Design-based logistic regression with log-transformed physical activity and sitting time tested the ratio constraint. **Results:** Each 1-standard-deviation increase in log-transformed PASTBI was associated with lower odds of metabolic syndrome (odds ratio [OR] 0.87, 95% confidence interval [CI] 0.84–0.91) and depressive symptoms (OR 0.89, 95% CI 0.83–0.96), and both associations remained essentially unchanged after mutual adjustment. Among active participants, doubling physical activity (OR 0.91) and sitting time (OR 1.10) were reciprocally associated with metabolic syndrome (ratio constraint *p* = 0.972). For depressive symptoms, only sitting time was associated (ratio constraint *p* = 0.011). Among inactive participants (13%), whose index was always zero, each doubling of sitting time was associated with depressive symptoms (OR 1.74, 95% CI 1.33–2.28). **Conclusions:** PASTBI was a valid summary for metabolic syndrome among active adults but not for depressive symptoms or inactive adults. Ratio-based indices should be verified for each outcome and population.

## 1. Introduction

Insufficient physical activity and prolonged sedentary behavior are well-established major risk factors for chronic diseases [1,2]. In 2020, the World Health Organization (WHO) for the first time recommended increasing physical activity and reducing sedentary time together, emphasizing the need to manage these two behaviors in an integrated manner rather than independently [3]. Because the two behaviors compete for limited time within a day, evaluating either one alone is insufficient to fully capture health risks [4]. Evidence that high levels of physical activity can substantially offset the mortality risk associated with prolonged sitting has further supported the importance of considering the balance between the two behaviors [5].

Physical activity and sedentary behavior influence both cardiometabolic and mental health. Longer sitting time has been associated with a higher risk of metabolic syndrome [6]. Regular physical activity has been shown to lower the risk of developing depression, and sitting time itself is positively associated with depressive symptoms [7,8]. Moreover, these two health outcomes are not independent of each other. Individuals with metabolic syndrome have a higher risk of depressive symptoms [9], and conversely, those with depression have an elevated risk of metabolic syndrome, indicating a bidirectional relationship [10]. However, the pathways leading to each outcome may differ. Energy expenditure and lipid metabolism are considered primary mechanisms linking these behaviors to cardiometabolic health [4], whereas factors accompanying the sedentary state itself, such as social isolation or television viewing, are thought to play a greater role in mental health outcomes [11].

Korea provides a relevant setting in which to examine these associations. Adult sitting time has continued to rise amid changes in occupational environments and the spread of screen-based leisure activities [12]. At the same time, the prevalence of metabolic syndrome increased from 27.1% in 2001 to 33.2% in 2020, and depressive disorders remain a major contributor to disease burden that requires continued management [13,14].

Several analytical approaches have been proposed to evaluate the combined effects of physical activity and sedentary behavior, including isotemporal substitution models [15] and compositional data analysis [16]. These methods appropriately account for the interdependence of time-use behaviors [17], yet they require specialized knowledge for calculation and interpretation, limiting their practical application in clinical or community health settings. To address this limitation, a simple ratio index, the physical activity–sitting time balance index (PASTBI), was recently proposed. The index is calculated by dividing weekly physical activity volume by daily sitting time and has been linked to all-cause mortality risk [18]. Nevertheless, evidence regarding PASTBI remains limited. Few studies have examined associations of the index with health outcomes other than mortality, and investigations in Asian populations, whose physical activity levels and sedentary patterns differ from those in Western countries, are scarce. In addition, a ratio index implicitly assumes that a proportional increase in physical activity and an equal proportional decrease in sitting time carry the same weight. This assumption can be tested directly, but it has not been examined. If the two behaviors contribute asymmetrically, the index may lose relevant information. Moreover, the index equals zero for everyone who reports no physical activity, so it cannot distinguish inactive individuals who sit for different lengths of time.

The present study aimed to investigate the associations of PASTBI with metabolic syndrome and depressive symptoms among Korean adults using data from the Korea National Health and Nutrition Examination Survey (KNHANES) 2014–2024. We further examined whether each association persisted after adjustment for the other outcome, whether the ratio constraint underlying the index held for each outcome, including among participants without physical activity, and which components of metabolic syndrome contributed most to the observed associations.

## 2. Materials and Methods

### 2.1. Study Design and Participants

This study used data from the KNHANES, a nationwide survey conducted annually by the Korea Disease Control and Prevention Agency (KDCA) under Article 16 of the National Health Promotion Act and designated as official government statistics (approval number 117002) [19,20]. The survey targets the non-institutionalized population residing in South Korea and selects participants through a stratified, multistage, clustered probability sampling design with survey districts as primary sampling units (PSUs) [19]. Data are collected throughout each calendar year through a health interview and health behavior questionnaire and a health examination at mobile examination centers, followed by a face-to-face nutrition survey in participants’ homes. Participation rates, defined as participation in at least one survey component, were 78.3%, 76.6%, 74.0%, and 69.4% for the 2013–2015, 2016–2018, 2019–2021, and 2022–2024 survey cycles, respectively [21,22]. In 2020, data collection was temporarily suspended because of the COVID-19 pandemic, and the health interview and examination were completed in 180 of the 192 sampled survey districts (93.8%) [21]. This study used public-use data files.

Because the Patient Health Questionnaire-9 (PHQ-9) was administered every other year, we pooled data from the survey years in which physical activity, sitting time, and PHQ-9 were collected simultaneously: 2014, 2016, 2018, 2020, 2022, and 2024.

**Figure 1:**
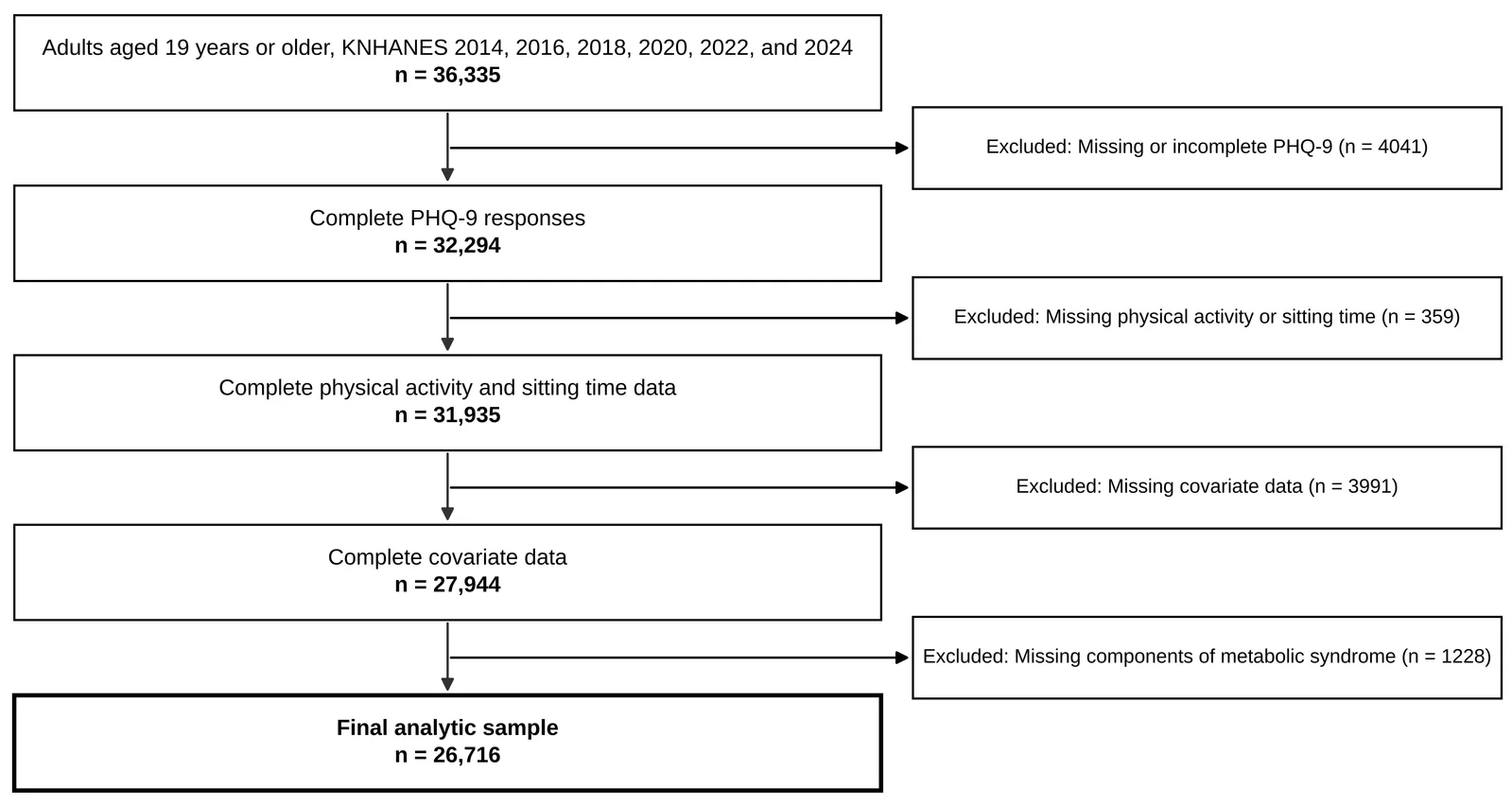
Flow of participant selection. KNHANES, Korea National Health and Nutrition Examination Survey; PHQ-9, Patient Health Questionnaire-9.

Of the 36,335 adults aged 19 years or older, 4041 had missing responses on any of the nine PHQ-9 items, 359 had missing physical activity or sitting time data, 3991 had missing covariates, and 1228 had missing metabolic syndrome components. Most participants with missing covariates (3480 of 3991) lacked only energy intake because they did not complete the nutrition survey. The final analytic sample comprised 26,716 participants (Figure 1).

Reporting follows the Preferred Reporting Items for Complex Sample Survey Analysis (PRICSSA).

### 2.2. Physical Activity–Sitting Time Balance Index

Physical activity was assessed with the Global Physical Activity Questionnaire (GPAQ) developed by the WHO [23], which has been included in KNHANES since 2014. Participants reported the number of days per week and the duration per day of vigorous-intensity and moderate-intensity activity in the work and leisure domains. Walking was assessed with a separate item on the number of days and the duration of walking for at least 10 min at a time, including walking for commuting, transport, and exercise. To avoid counting the same walking twice, the GPAQ transport domain, which also covers walking for travel, was not added to the total. Because brisk walking at work or during leisure could also have been reported as moderate-intensity activity, some overlap between the walking item and the moderate-intensity domains cannot be excluded. Weekly physical activity volume was calculated as the sum of days, minutes, and metabolic equivalent of task (MET) values across activity types. MET values of 8.0 and 4.0 were assigned to vigorous and moderate activity according to the GPAQ protocol [23]. Because the walking item follows the format of the International Physical Activity Questionnaire, a value of 3.3 METs was assigned to walking according to its scoring protocol [24]. The robustness of this definition was examined in sensitivity analyses described below.

Sitting time was obtained from the question on the average time spent sitting or reclining per day, excluding sleep, and was converted to hours per day.

PASTBI was calculated according to the method proposed by Botlero and colleagues [18] by dividing weekly physical activity volume (MET-min/week) by daily sitting time (hours/day). Higher values indicate a greater volume of physical activity relative to time spent sitting. Because the distribution was highly right-skewed, PASTBI was log-transformed as log(1 + PASTBI) and standardized when analyzed as a continuous variable. Participants were also divided into quartiles. Under log transformation, the choice of time units in the numerator and denominator changes the index only by a constant and does not affect any estimate.

### 2.3. Metabolic Syndrome

Metabolic syndrome was defined according to the revised National Cholesterol Education Program Adult Treatment Panel III criteria [25] as three or more of the following five components. Abdominal obesity was defined as a waist circumference of 90 cm or greater in men and 85 cm or greater in women, according to the Korean-specific cut-offs of the Korean Society for the Study of Obesity [26]. Elevated blood pressure was defined as systolic blood pressure of 130 mmHg or higher, diastolic blood pressure of 85 mmHg or higher, or use of antihypertensive medication. Elevated fasting glucose was defined as 100 mg/dL or higher or use of antidiabetic medication. Elevated triglycerides were defined as 150 mg/dL or higher and reduced high-density lipoprotein cholesterol (HDL-C) as less than 40 mg/dL in men and less than 50 mg/dL in women. The number of components present (0 to 5) was also counted for each participant.

### 2.4. Depressive Symptoms

Depressive symptoms were assessed with the Korean version of the PHQ-9, which has demonstrated validity [27]. The PHQ-9 consists of nine items on the frequency of depressive symptoms over the past two weeks, each scored from 0 to 3, yielding a total score from 0 to 27 [28]. Clinically relevant depressive symptoms were defined as a score of 10 or higher, a threshold identified as optimal in a previous meta-analysis [29].

### 2.5. Covariates

Covariates were selected on the basis of their known associations with the exposure and outcomes. They included age, sex, survey year, education level (elementary school or lower, middle school, high school, college or higher), household income quartile, marital status (married or cohabiting versus other), occupation (white-collar, service or sales, manual or agricultural, unemployed or economically inactive), smoking status (never, former, current), frequency of alcohol consumption, daily energy intake, and body mass index. Self-rated health and perceived stress were not adjusted because they are more likely to reflect depressive symptoms or to lie on the causal pathway than to confound the association [30].

### 2.6. Statistical Analysis

All analyses accounted for the complex sample design. Because the analysis included variables from the health interview, health examination, and nutrition survey, the combined weight for all three components was used and divided by six for the pooled survey years [19]. The KNHANES weights incorporate design weights, adjustment for household and individual nonresponse, and post-stratification to annual population estimates, which partly compensate for differences in participation across survey years [22]. Variance was estimated with Taylor series linearization using variance estimation strata, made unique within each survey year (160 strata), and PSUs (1140 PSUs), giving 980 design degrees of freedom. No stratum contained a single PSU. The survey design was defined for all adults and estimates for the analytic sample and for subgroups were obtained by domain estimation rather than by deleting observations [31]. Complete-case analysis was used because missing data arose mainly from nonparticipation in a survey component rather than from scattered item nonresponse, and because odds ratios from complete-case analysis are not biased when missingness does not depend on the outcome after conditioning on the exposure and covariates. Reporting followed the Preferred Reporting Items for Complex Sample Survey Analysis (PRICSSA) [32] (Table S6).

Participant characteristics are presented as unweighted numbers with weighted percentages, weighted means with standard errors, or weighted medians with interquartile ranges. Differences across quartiles were tested with design-based Wald tests, using log-transformed values for skewed variables.

Associations of PASTBI with metabolic syndrome and depressive symptoms were estimated with design-based logistic regression and expressed as odds ratios (ORs) and 95% confidence intervals (CIs). The highest quartile served as reference. Linear trend across quartiles was tested with an orthogonal polynomial contrast of the quartile coefficients and, alternatively, by entering the median value of each quartile as a continuous variable. ORs per 1-standard-deviation increase in log-transformed PASTBI were also estimated. Dose–response relationships were examined with restricted cubic splines, with the weighted median PASTBI as the reference. Knots were placed at the 5th, 27.5th, 50th, 72.5th, and 95th percentiles [33]. The number of knots was chosen by comparing the Akaike information criterion of models with three, four, and five knots, and five knots provided the best fit for both outcomes (Table S3). Overall and nonlinear associations were tested with design-based Wald tests.

To assess whether each association persisted after accounting for the other outcome, each outcome model was additionally adjusted for the other outcome, and the proportional attenuation of the regression coefficient was calculated. Analyses stratified by the presence of the other outcome and tests of multiplicative interaction were also performed.

For participants with any physical activity, PASTBI is positive and log(PASTBI) equals log(physical activity) minus log(sitting time) exactly. The ratio form therefore constrains the coefficients of the two log-transformed behaviors to be equal in magnitude and opposite in sign. This identity does not hold when physical activity is zero, which applied to 13.0% of participants. For this reason, the main continuous exposure was log(1 + PASTBI), and the constraint test in the full sample entered log(1 + physical activity) and log(sitting time) in the same model and applied a Wald test to the sum of the two coefficients. Because the offset makes this full-sample test an approximation, the test was repeated among participants with any physical activity using log(physical activity) without an offset, which is the exact form of the constraint, and the sensitivity of the full-sample test to offsets of 10 and 100 MET-min/week was examined (Table S2). ORs are presented per doubling of each exposure. Because participants without physical activity have an index of zero regardless of sitting time, the association of sitting time with each outcome was also estimated in this group. Variance inflation factors for the two log-transformed behaviors were 1.08 and 1.16, and their correlation was −0.04. PASTBI was not entered together with its two components because it is a deterministic function of them. The share of the variance of the log-transformed index attributable to each behavior was calculated from their variances, given their near-zero correlation.

Subgroup analyses were performed by sex and age group (19–39, 40–64, 65–74, and 75 years or older), and interactions were evaluated with design-based Wald tests of product terms. Sensitivity analyses included exclusion of PASTBI values above the 99th percentile, an alternative PHQ-9 cut-off of 5 or higher, outcome-specific covariate sets, unweighted models, and an enlarged sample that omitted energy intake as a covariate and used the health interview and examination weight. The outcome-specific sets excluded marital status, occupation, and body mass index from the metabolic syndrome model and energy intake from the depressive-symptom model. To assess the influence of the COVID-19 pandemic and of the physical activity definition, the main analyses and the ratio constraint test were repeated after excluding the 2020 and 2022 survey years, with physical activity calculated by standard GPAQ scoring of the work, transport, and leisure domains without the separate walking item, and with physical activity excluding the walking item.

Analyses were performed with R version 4.3.3 (R Foundation for Statistical Computing, Vienna, Austria) using the survey and rms packages. A two-sided *p* value less than 0.05 was considered statistically significant. Claude Opus 5.5 (Anthropic) was used to assist with statistical programming and with drafting and editing of the manuscript text. All code, outputs, and text were checked by the authors.

## 3. Results

### 3.1. Participant Characteristics

Table 1 presents the characteristics of the 26,716 participants. The weighted mean age was 47.3 years, and 49.7% were women. The weighted median PASTBI was 101 (interquartile range 35–248), the weighted median physical activity volume was 815 MET-min/week, and the weighted mean sitting time was 8.5 h per day. A total of 3465 participants (13.0%) reported no physical activity.

Compared with participants in the highest PASTBI quartile, those in the lowest quartile were older (49.6 versus 46.2 years), sat longer (9.6 versus 6.3 h per day), and accumulated much less physical activity (median 66 versus 2799 MET-min/week). They were also more likely to have an elementary-school education or lower (18.1% versus 9.1%) and to be in the lowest household income quartile (18.1% versus 11.5%). The prevalence of metabolic syndrome decreased from 30.6% in the lowest quartile to 21.8% in the highest quartile, and the mean number of metabolic risk factors decreased from 1.75 to 1.44 (Table S5). The prevalence of depressive symptoms was 6.1% in the lowest quartile and 4.3% in the highest quartile (all *p* < 0.001).

### 3.2. Association of PASTBI with Metabolic Syndrome and Depressive Symptoms

PASTBI showed a graded inverse association with metabolic syndrome (Table 2). Compared with the highest quartile, the odds of metabolic syndrome were 46% and 47% higher in the lowest and second quartiles (OR 1.46, 95% CI 1.31–1.63, and OR 1.47, 95% CI 1.32–1.64) and 12% higher in the third quartile (OR 1.12, 95% CI 1.01–1.26), with a significant linear trend (*p* < 0.001). Each 1-standard-deviation increase in log-transformed PASTBI was associated with 13% lower odds of metabolic syndrome (OR 0.87, 95% CI 0.84–0.91).

For depressive symptoms, the odds were 29% higher in the lowest quartile than in the highest quartile (OR 1.29, 95% CI 1.07–1.55), whereas the second and third quartiles did not differ from the reference. The linear trend was significant with the orthogonal contrast (*p* = 0.032) but not with the median-score test (*p* = 0.114). Each 1-standard-deviation increase in log-transformed PASTBI was associated with 11% lower odds of depressive symptoms (OR 0.89, 95% CI 0.83–0.96).

### 3.3. Dose–Response Relationship

**Figure 2:**
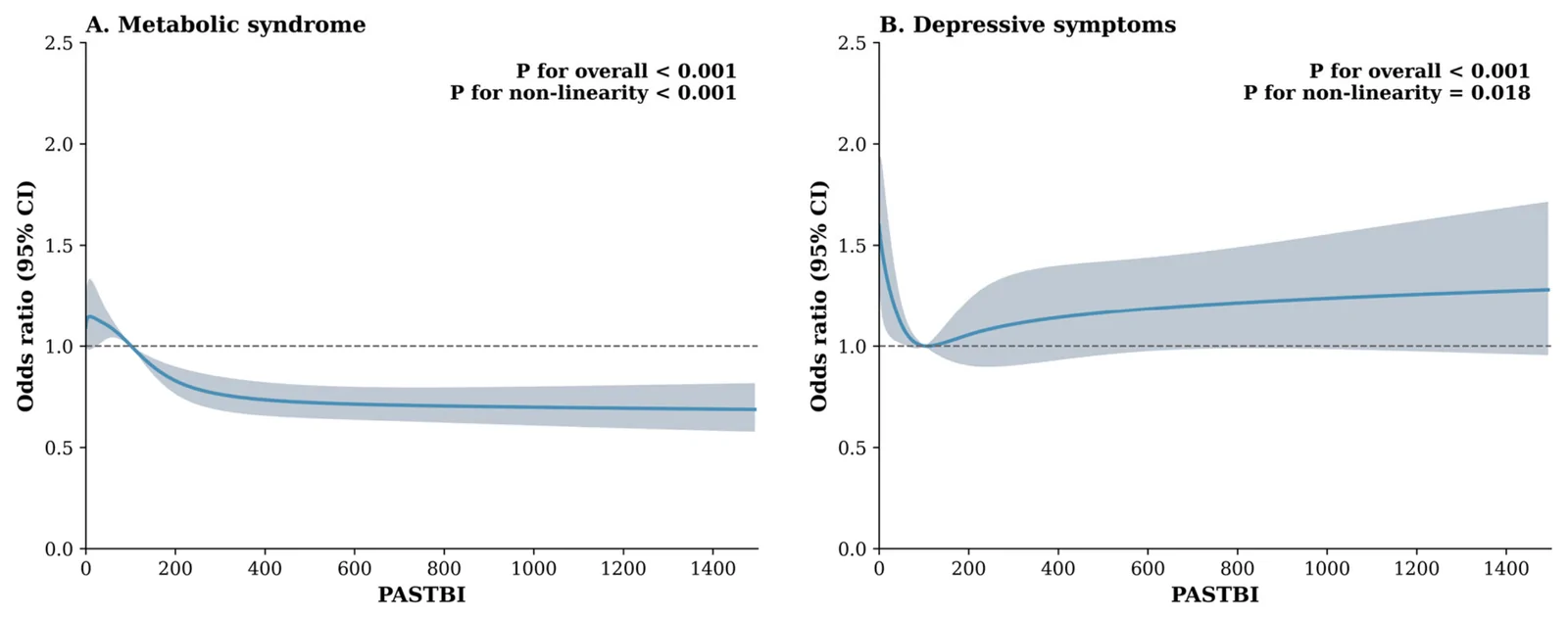
Dose–response associations of the physical activity–sitting time balance index (PASTBI) with (**A**) metabolic syndrome and (**B**) depressive symptoms. Lines represent odds ratios and shaded areas 95% confidence intervals. The weighted median PASTBI (101) was used as the reference, and the x-axis is truncated at 1500.

For metabolic syndrome, both the overall and the nonlinear associations were significant (*p* < 0.001 for both). The odds decreased as PASTBI increased up to approximately the median and changed little thereafter (Figure 2A). For depressive symptoms, the overall association was significant (*p* < 0.001), and the test for nonlinearity was also significant (*p* = 0.018). Elevated odds were confined to the lowest range of PASTBI, with no further reduction above the median (Figure 2B). Models with three or four knots yielded the same conclusions (Table S3).

### 3.4. Mutual Adjustment and Stratified Analyses

Additional adjustment for depressive symptoms left the OR for metabolic syndrome at 0.87 (95% CI 0.84–0.91), an attenuation of 0.8% (Table 3). Additional adjustment for metabolic syndrome left the OR for depressive symptoms at 0.90 (95% CI 0.84–0.96), an attenuation of 2.8%. Both associations therefore remained essentially unchanged after mutual adjustment. The association of PASTBI with depressive symptoms was stronger among participants with metabolic syndrome (OR 0.81, 95% CI 0.71–0.92) than among those without (OR 0.94, 95% CI 0.86–1.02), but the interaction was not significant (*p* = 0.194). The interaction between PASTBI and depressive symptoms in the metabolic syndrome model was also not significant (*p* = 0.497).

### 3.5. Test of the Ratio Constraint

On the log scale, the standard deviation of physical activity was about five times that of sitting time (2.50 versus 0.50), and physical activity accounted for 96.2% of the variance of the log-transformed index. Table 4 shows the associations of the two behaviors with each outcome. In the full sample, the ratio constraint was rejected for both metabolic syndrome (*p* = 0.009) and depressive symptoms (*p* < 0.001).

Among the 23,251 participants with any physical activity, the ORs for metabolic syndrome per doubling were 0.91 (95% CI 0.89–0.94) for physical activity and 1.10 (95% CI 1.03–1.17) for sitting time. These estimates were nearly reciprocal, and the ratio constraint was not rejected (*p* = 0.972). For depressive symptoms, physical activity was not associated with the outcome (OR 1.01, 95% CI 0.96–1.06), whereas sitting time was (OR 1.20, 95% CI 1.05–1.36), and the constraint was rejected (*p* = 0.011).

Among the 3465 participants without physical activity, each doubling of sitting time was associated with 74% higher odds of depressive symptoms (OR 1.74, 95% CI 1.33–2.28). The corresponding estimate for metabolic syndrome was similar in direction to that in active participants but was not statistically significant (OR 1.09, 95% CI 0.95–1.25). For metabolic syndrome, the result of the constraint test in the full sample depended on the offset added to zero physical activity, whereas the rejection for depressive symptoms was consistent across offsets (Table S2).

### 3.6. Individual Components of Metabolic Syndrome

Each 1-standard-deviation increase in log-transformed PASTBI was associated with 19% lower odds of abdominal obesity (OR 0.81, 95% CI 0.78–0.86) and 10% lower odds of both elevated triglycerides (OR 0.90, 95% CI 0.87–0.94) and reduced HDL-C (OR 0.90, 95% CI 0.87–0.93) (Table 5). The association with elevated fasting glucose was weak (OR 0.97, 95% CI 0.94–1.00, *p* = 0.041), and that with elevated blood pressure was not statistically significant (OR 1.00, 95% CI 0.96–1.03).

### 3.7. Sensitivity and Subgroup Analyses

The main findings were similar in all sensitivity analyses (Table S1). Excluding PASTBI values above the 99th percentile (*n* = 26,448) yielded ORs of 0.87 for metabolic syndrome and 0.88 for depressive symptoms. A PHQ-9 cut-off of 5 or higher preserved the association with depressive symptoms (OR 0.92, 95% CI 0.88–0.96). Outcome-specific covariate sets, unweighted models, and the enlarged sample of 29,998 participants without energy intake produced ORs between 0.88 and 0.90 for both outcomes. Excluding the 2020 and 2022 survey years did not change the conclusions (Table S8). The association with metabolic syndrome and the ratio constraint test results were also consistent when physical activity was calculated by standard GPAQ scoring or without the walking item. The association between PASTBI and depressive symptoms weakened under these alternative definitions (OR 0.93 and 1.05), which increased the proportion of participants with an index of zero to 29.5% and 62.3%, respectively. In these participants, sitting time remained associated with depressive symptoms (OR per doubling 1.53 and 1.45). In subgroup analyses by sex and age group, none of the interaction tests was significant (all *p* > 0.3) (Table S4).

## 4. Discussion

This study examined the associations of PASTBI with metabolic syndrome and depressive symptoms in Korean adults. PASTBI was inversely associated with both outcomes, and these associations remained essentially unchanged after mutual adjustment for the other outcome. A direct test of the ratio constraint showed that the index behaved differently across outcomes and populations. Among physically active adults, physical activity and sitting time contributed reciprocally to metabolic syndrome, as the ratio form assumes. For depressive symptoms, sitting time alone carried the association. In addition, the index assigned a value of zero to all inactive adults and thereby discarded the strongest association observed for depressive symptoms, which involved sitting time in this group.

The association between PASTBI and metabolic syndrome was consistent in direction with a previous study that used mortality as the outcome [18]. The index therefore appears to capture not only the distal endpoint of death but also the accumulation of metabolic risk factors that precede it. The shape of the dose–response curve also aligns with existing evidence. In a cohort of Taiwanese adults, as little as 92 min of activity per week, or approximately 15 min per day, was associated with a 14% lower mortality risk compared with inactivity, and each additional 15 min conferred only a further 4% reduction [34]. A meta-analysis of non-occupational physical activity similarly showed that roughly half of the benefit was achieved at 8.75 MET-hours per week [35]. In the present study, the odds of metabolic syndrome decreased up to approximately the median PASTBI and changed little thereafter. This pattern suggests that modest improvements among those with the least favorable balance may yield the greatest public-health gain.

In component-specific analyses, the clearest associations were observed for abdominal obesity, elevated triglycerides, and reduced HDL-C, whereas the association with fasting glucose was weak and that with blood pressure was absent. Prolonged muscle inactivity suppresses lipoprotein lipase activity in skeletal muscle, thereby reducing triglyceride clearance and HDL production [36]. Experimental studies in which sitting was interrupted by brief activity bouts have demonstrated improvements in postprandial metabolic responses [37]. The relatively direct involvement of lipid pathways has also been supported in longitudinal research. Increases in sitting time were associated with higher triglycerides and clustered metabolic risk, yet these relationships were not mediated by changes in waist circumference or body mass index [38]. The association with abdominal obesity persisted after adjustment for body mass index. Sedentary behavior may therefore influence abdominal fat distribution through pathways distinct from overall adiposity, which is consistent with a report that each 15-min daily increase in sitting time over six years was linked to a 0.13 cm increase in waist circumference [39]. Blood pressure and glucose, however, are strongly influenced by pharmacologic treatment. In this study, treated individuals were classified as having the respective risk factors. Because medically controlled values do not necessarily reflect current behavioral patterns, associations may have been attenuated. The inconsistent relationship between sedentary behavior and blood pressure reported in a prior meta-analysis [40] is in line with this interpretation.

Findings for depressive symptoms differed in part from previous literature. Although prospective evidence that physical activity protects against incident depression is relatively well established [7], physical activity was not associated with depressive symptoms among active participants once sitting time was considered. The GPAQ captures only moderate-to-vigorous activity lasting at least 10 min and therefore misses light-intensity activity. However, a meta-analysis of isotemporal substitution studies found no significant benefit when sitting was replaced by light activity, whereas replacement with moderate-to-vigorous activity reduced depressive symptoms [41]. Measurement limitations alone therefore appear insufficient to account for this finding. A more likely explanation lies in the type of sedentary behavior. The mental health impact of sitting appears to depend more on the nature of the activity than on total volume. Passive screen-based sitting has been linked to a higher risk of depression, whereas cognitively active sitting, such as reading or desk work, has not [42,43]. The sitting-time question used in this study did not distinguish among types of sedentary behavior. The association observed here may therefore partly reflect passive sitting and the accompanying social isolation. Study design may also have contributed. Mendelian randomization analyses have provided causal evidence that physical activity lowers depression risk while finding little support for the reverse pathway [44]. In a cross-sectional design the two directions can become entangled, which may lead to underestimation of the association with activity. The concentration of elevated odds in the lowest PASTBI range is consistent with reports that the relationship between sitting time and depression risk becomes evident only beyond a certain level [45].

Chronic inflammation and activation of the hypothalamic–pituitary–adrenal axis have been proposed as shared mechanisms linking metabolic dysregulation and depressive symptoms [46]. If these common pathways were the primary mediators, adjustment for metabolic syndrome should have substantially attenuated the association with depressive symptoms. In the present study, however, attenuation was minimal and the interaction was not significant. This finding implies that physical activity and sedentary behavior may act on mental health through pathways that are at least partly distinct from metabolic routes. Replacement of sitting with moderate-intensity activity has been accompanied by improvements in inflammatory markers [47]. Even so, the reduction in social interaction and environmental stimulation that accompanies passive sitting may operate independently of metabolic indicators.

The structure of the index explains many of these findings. Because physical activity varied about five times more than sitting time on the log scale, the ranking of participants by PASTBI was determined almost entirely by physical activity. This dominance does not arise from the different time units of the numerator and denominator, which only rescale the index by a constant. It reflects the much wider population variation in physical activity. Where the ratio constraint holds, this dominance is not a defect. Among active adults, a doubling of physical activity and a halving of sitting time were associated with metabolic syndrome to the same degree, so the index correctly weighted both behaviors even though its variation came mainly from physical activity. Where the constraint fails, the same dominance becomes a problem. For depressive symptoms, the behavior that carried the association contributed little to the variation of the index, and the index therefore diluted the signal from sitting time. The zero value assigned to all inactive adults is a further structural limitation. In this group, which made up 13% of the sample, sitting time showed its strongest association with depressive symptoms, yet the index could not reflect it. The sensitivity analyses illustrate the same point. When narrower definitions of physical activity increased the proportion of participants with an index of zero, the association between the index and depressive symptoms weakened, whereas sitting time remained associated with depressive symptoms in those participants.

By applying PASTBI simultaneously to cardiometabolic and mental-health outcomes, the present study shows that the index is associated with intermediate health endpoints as well as with mortality. More importantly, it provides a formal test of the assumption that underlies any ratio-based behavioral index and identifies where that assumption holds and where it fails. These findings are consistent with substitution analyses showing that reductions in sitting time and increases in activity are not equivalent for mental-health outcomes [41]. From a practical standpoint, the simplicity of PASTBI may make it a potentially useful summary indicator of cardiometabolic health among physically active adults in community or primary-care settings, pending prospective validation of its predictive value. When mental health is the focus, or when a substantial proportion of the target population is inactive, sitting time should be assessed separately. Given reports that the relationship between metabolic syndrome and depressive symptoms is less consistent in Asian than in Western populations [9], this study also adds evidence from an Asian population.

The cross-sectional design precludes causal inference. Reverse causation is plausible, particularly for depressive symptoms, which can reduce physical activity and increase sitting time. Exposure variables were self-reported. Self-reported physical activity shows only moderate correlation with accelerometer measures and tends to be over-reported [48], while sitting time tends to be under-reported [49]. Such measurement error may bias estimates in either direction, particularly because errors in physical activity and sitting time may be correlated and may differ by health status. Depressive symptoms were assessed with a screening instrument. Although the PHQ-9 is validated, it does not replace clinical diagnosis. Approximately one-quarter of eligible adults were excluded because of missing data. Excluded individuals were older, less educated, and less physically active, and had a higher prevalence of depressive symptoms (7.0% versus 5.0%) and metabolic syndrome (32.9% versus 27.3%) (Table S7). Because exclusion was related to both exposure and outcomes, the direction of any resulting selection bias cannot be determined. However, results were similar in an enlarged sample that retained participants who did not complete the nutrition survey. Participation in KNHANES declined across the survey cycles. Although the survey weights adjust for nonresponse and post-stratify to population totals, residual nonresponse bias cannot be excluded if nonparticipation was related to physical activity, sitting time, or health status. The 2020 and 2022 surveys were conducted during and after the COVID-19 pandemic, which may have altered physical activity, sitting time, and depressive symptoms, but the main conclusions were unchanged when these years were excluded.

Several features of the analysis also warrant caution in interpretation. Because ORs can overestimate risk ratios when the outcome is common, the ORs for metabolic syndrome should not be read directly as relative risks. Residual confounding by unmeasured factors such as sleep duration, diet quality, and antidepressant use cannot be ruled out. Sleep forms part of the 24 h time-use composition together with physical activity and sitting, and an index that omits sleep captures only a portion of daily behavior. The constraint test in the full sample required an offset for zero physical activity, and its result for metabolic syndrome depended on that choice. We therefore based our interpretation on participants with any physical activity, in whom no offset was needed.

Future studies using accelerometer-based measurement and prospective designs are needed to clarify causal direction. Distinguishing types of sedentary behavior would allow more precise characterization of relationships with mental health. For outcomes in which the two behaviors contribute asymmetrically, and for populations with many inactive individuals, weighted indices or separate assessment of each behavior may be more appropriate.

## 5. Conclusions

Among Korean adults, PASTBI was associated with both metabolic syndrome and depressive symptoms, and both associations remained essentially unchanged after mutual adjustment. The ratio constraint underlying the index held for metabolic syndrome among physically active adults but not for depressive symptoms, and the index discarded information on sitting time among inactive adults. Ratio-based behavioral indices should be tested against their constituent behaviors for each target outcome and population, and their predictive value should be established in prospective studies before they are used in practice.

## Figures and Tables

**Table 1:** Participant characteristics according to quartiles of the physical activity–sitting time balance index.

Characteristic	Total	Q1 (≤33)	Q2 (33–99)	Q3 (99–238)	Q4 (>238)	*p*
No. of participants	26,716	7050	6583	6408	6675	
PASTBI, median (IQR)	101 (35–248)	7 (0–21)	63 (50–80)	154 (124–192)	445 (313–715)	<0.001
Physical activity, MET-min/week, median (IQR)	815 (297–1806)	66 (0–198)	594 (396–792)	1350 (924–1626)	2799 (1983–4177)	<0.001
Sitting time, h/day	8.5 ± 0.03	9.6 ± 0.06	9.7 ± 0.05	8.6 ± 0.05	6.3 ± 0.05	<0.001
Age, years	47.3 ± 0.19	49.6 ± 0.29	46.6 ± 0.29	46.7 ± 0.28	46.2 ± 0.25	<0.001
Women	15,331 (49.7)	4153 (51.0)	4009 (53.5)	3713 (50.3)	3456 (44.4)	<0.001
Education						<0.001
Elementary school or lower	4828 (12.1)	1844 (18.1)	1180 (11.8)	906 (9.6)	898 (9.1)	
Middle school	2683 (8.0)	822 (9.9)	615 (7.5)	567 (6.9)	679 (7.6)	
High school	8936 (36.4)	2158 (34.3)	2117 (34.8)	2178 (36.1)	2483 (40.2)	
College or higher	10,269 (43.5)	2226 (37.7)	2671 (45.9)	2757 (47.4)	2615 (43.1)	
Household income						<0.001
Lowest quartile	4717 (14.0)	1616 (18.1)	1178 (14.2)	985 (12.1)	938 (11.5)	
Second quartile	6502 (23.0)	1775 (24.0)	1563 (22.4)	1532 (22.7)	1632 (23.1)	
Third quartile	7561 (30.1)	1898 (29.3)	1842 (29.8)	1866 (30.9)	1955 (30.4)	
Highest quartile	7936 (32.9)	1761 (28.6)	2000 (33.5)	2025 (34.3)	2150 (35.1)	
Occupation						<0.001
White-collar	6700 (29.1)	1622 (27.3)	1940 (34.6)	1760 (31.0)	1378 (23.6)	
Service or sales	4681 (16.7)	1354 (17.4)	969 (14.0)	1047 (15.8)	1311 (19.6)	
Manual or agricultural	4949 (19.0)	1392 (21.3)	1100 (15.9)	1049 (16.3)	1408 (22.3)	
Unemployed or economically inactive	10,386 (35.2)	2682 (33.9)	2574 (35.5)	2552 (36.8)	2578 (34.5)	
Married or cohabiting	21,951 (75.1)	6070 (80.4)	5288 (73.0)	5143 (72.8)	5450 (74.1)	<0.001
Smoking status						<0.001
Never	16,490 (57.3)	4225 (54.3)	4233 (60.1)	4100 (60.1)	3932 (54.9)	
Former	5919 (23.4)	1480 (22.5)	1399 (22.6)	1422 (23.6)	1618 (24.6)	
Current	4307 (19.3)	1345 (23.1)	951 (17.3)	886 (16.3)	1125 (20.4)	
Body mass index, kg/m^2^	24.1 ± 0.03	24.1 ± 0.06	24.0 ± 0.06	24.0 ± 0.05	24.1 ± 0.06	0.050
Energy intake, kcal/day	1979 ± 8	1915 ± 13	1930 ± 13	1991 ± 14	2077 ± 15	<0.001
No. of metabolic risk factors	1.55 ± 0.01	1.75 ± 0.02	1.57 ± 0.02	1.43 ± 0.02	1.44 ± 0.02	<0.001
Metabolic syndrome	7302 (25.4)	2306 (30.6)	1902 (26.9)	1528 (22.6)	1566 (21.8)	<0.001
PHQ-9 score, median (IQR)	1 (0–3)	1 (0–4)	1 (0–3)	1 (0–3)	1 (0–3)	0.003
Depressive symptoms	1332 (4.8)	451 (6.1)	294 (4.3)	289 (4.4)	298 (4.3)	<0.001

Values are unweighted n (weighted %), weighted mean ± standard error (SE), or weighted median (interquartile range, IQR). MET, metabolic equivalent of task; PASTBI, physical activity–sitting time balance index; PHQ-9, Patient Health Questionnaire-9.

**Table 2:** Odds ratios for metabolic syndrome and depressive symptoms according to quartiles of the physical activity–sitting time balance index.

Outcome and PASTBI Quartile	No. of Participants	No. of Cases	Weighted Prevalence, %	OR (95% CI)	*p*
**Metabolic syndrome**					
Q1 (≤33)	7050	2306	30.6	1.46 (1.31–1.63)	<0.001
Q2 (33–99)	6583	1902	26.9	1.47 (1.32–1.64)	<0.001
Q3 (99–238)	6408	1528	22.6	1.12 (1.01–1.26)	0.038
Q4 (>238)	6675	1566	21.8	1.00 (reference)	
P for trend					<0.001
**Depressive symptoms**					
Q1 (≤33)	7050	451	6.1	1.29 (1.07–1.55)	0.007
Q2 (33–99)	6583	294	4.3	0.92 (0.76–1.12)	0.420
Q3 (99–238)	6408	289	4.4	1.03 (0.84–1.25)	0.784
Q4 (>238)	6675	298	4.3	1.00 (reference)	
P for trend					0.032

The highest quartile served as the reference. CI, confidence interval; OR, odds ratio; PASTBI, physical activity–sitting time balance index.

**Table 3:** Mutually adjusted and stratified associations of the physical activity–sitting time balance index with metabolic syndrome and depressive symptoms.

Outcome and Model	No. of Participants	No. of Cases	OR (95% CI)	*p*
**Metabolic syndrome**				
Main model	26,716	7302	0.87 (0.84–0.91)	<0.001
Additionally adjusted for depressive symptoms	26,716	7302	0.87 (0.84–0.91)	<0.001
Attenuation of the coefficient, %			0.8	
Stratified: depressive symptoms absent	25,384	6900	0.88 (0.84–0.91)	<0.001
Stratified: depressive symptoms present	1332	402	0.81 (0.69–0.96)	0.014
P for interaction				0.497
**Depressive symptoms**				
Main model	26,716	1332	0.89 (0.83–0.96)	0.002
Additionally adjusted for metabolic syndrome	26,716	1332	0.90 (0.84–0.96)	0.002
Attenuation of the coefficient, %			2.8	
Stratified: metabolic syndrome absent	19,414	930	0.94 (0.86–1.02)	0.138
Stratified: metabolic syndrome present	7302	402	0.81 (0.71–0.92)	<0.001
P for interaction				0.194

Odds ratios correspond to a 1-standard deviation (SD) increase in log-transformed PASTBI. Attenuation denotes the proportional change in the regression coefficient after additional adjustment for the other outcome. CI, confidence interval; OR, odds ratio; PASTBI, physical activity–sitting time balance index.

**Table 4:** Associations of physical activity and sitting time with metabolic syndrome and depressive symptoms and test of the ratio constraint.

Outcome and Sample	No. of Participants	No. of Cases	Physical Activity OR (95% CI)	*P*	Sitting Time OR (95% CI)	*p*	P for Ratio Constraint
**Metabolic syndrome**							
All participants	26,716	7302	0.97 (0.96–0.98)	<0.001	1.12 (1.05–1.18)	<0.001	0.009
Participants with any physical activity	23,251	6063	0.91 (0.89–0.94)	<0.001	1.10 (1.03–1.17)	0.003	0.972
Participants without physical activity	3465	1239	—		1.09 (0.95–1.25)	0.232	—
**Depressive symptoms**							
All participants	26,716	1332	0.98 (0.96–0.99)	0.011	1.26 (1.13–1.42)	<0.001	<0.001
Participants with any physical activity	23,251	1089	1.01 (0.96–1.06)	0.716	1.20 (1.05–1.36)	0.006	0.011
Participants without physical activity	3465	243	—		1.74 (1.33–2.28)	<0.001	—

Odds ratios correspond to a doubling of each exposure, estimated with both exposures in the same model. The ratio constraint assumes that the coefficients for log-transformed physical activity and log-transformed sitting time are equal in magnitude and opposite in sign. PASTBI equals zero for all participants without physical activity. CI, confidence interval; OR, odds ratio; PASTBI, physical activity–sitting time balance index.

**Table 5:** Associations of the physical activity–sitting time balance index with individual components of metabolic syndrome.

Component	No. of Cases	Weighted Prevalence, %	OR (95% CI)	*p*
Abdominal obesity	8668	31.3	0.81 (0.78–0.86)	<0.001
Elevated triglycerides	7276	28.3	0.90 (0.87–0.94)	<0.001
Reduced HDL cholesterol	7522	25.9	0.90 (0.87–0.93)	<0.001
Elevated blood pressure	10,340	35.4	1.00 (0.96–1.03)	0.775
Elevated fasting glucose	9853	33.9	0.97 (0.94–1.00)	0.041

Odds ratios correspond to a 1-SD increase in log-transformed PASTBI. CI, confidence interval; HDL, high-density lipoprotein; OR, odds ratio; PASTBI, physical activity–sitting time balance index; SD, standard deviation.

## Data Availability

The data presented in this study are available in the Korea National Health and Nutrition Examination Survey repository at https://knhanes.kdca.go.kr (accessed on 1 October 2026). These data were derived from resources available in the public domain and can be downloaded free of charge after registration. The analytic code is available from the corresponding author upon reasonable request.

## Supplementary Materials

The following supporting information can be downloaded at https://www.mdpi.com/article/10.3390/healthcare14213714/s1, Table S1: Sensitivity analyses; Table S2: Test of the ratio constraint according to the handling of zero physical activity; Table S3: Restricted cubic spline models with different numbers of knots; Table S4: Associations of the physical activity–sitting time balance index with metabolic syndrome and depressive symptoms according to sex and age group; Table S5: Distribution of the number of metabolic risk factors according to quartiles of the physical activity–sitting time balance index; Table S6: Preferred Reporting Items for Complex Sample Survey Analysis (PRICSSA) checklist; Table S7: Characteristics of included and excluded participants; Table S8: Sensitivity analyses for the survey period and the definition of physical activity.

## Abbreviations

AIC, Akaike information criterion; CI, confidence interval; GPAQ, Global Physical Activity Questionnaire; HDL-C, high-density lipoprotein cholesterol; KDCA, Korea Disease Control and Prevention Agency; KNHANES, Korea National Health and Nutrition Examination Survey; MET, metabolic equivalent of task; OR, odds ratio; PASTBI, physical activity–sitting time balance index; PHQ-9, Patient Health Questionnaire-9; PRICSSA, Preferred Reporting Items for Complex Sample Survey Analysis; PSU, primary sampling unit; SD, standard deviation; WHO, World Health Organization.
