# Supplementary material for: Association of the Physical Activity–Sitting Time Balance Index with Metabolic Syndrome and Depressive Symptoms in Korean Adults: KNHANES 2014–2024
Source: Healthcare (Basel). 2026 Nov 2;14(21):3714. doi: 10.3390/healthcare14213714 (PMC13762572; doi:10.3390/healthcare14213714)
Supplement: Supplementary file 1 [file healthcare-14-03714-s001.zip › healthcare-4634886-supplementary.pdf]

Table S1. Sensitivity analyses

Analysis	Outcome	No. of participants	No. of cases	OR (95% CI)	P
Main analysis	Metabolic syndrome	26,716	7,302	0.87 (0.84–0.91)	<0.001
Main analysis	Depressive symptoms	26,716	1,332	0.89 (0.83–0.96)	0.002
Excluding PASTBI values above the 99th percentile	Metabolic syndrome	26,448	7,241	0.87 (0.84–0.91)	<0.001
Excluding PASTBI values above the 99th percentile	Depressive symptoms	26,448	1,317	0.88 (0.82–0.95)	<0.001
PHQ-9 cut-off of 5 or higher	Depressive symptoms (PHQ-9 ≥5)	26,716	4,921	0.92 (0.88–0.96)	<0.001
Outcome-specific covariate set	Metabolic syndrome	26,716	7,302	0.88 (0.85–0.91)	<0.001
Outcome-specific covariate set	Depressive symptoms	26,716	1,332	0.89 (0.83–0.96)	0.002
Unweighted model	Metabolic syndrome	26,716	7,302	0.90 (0.87–0.92)	<0.001
Unweighted model	Depressive symptoms	26,716	1,332	0.90 (0.86–0.95)	<0.001
Without energy intake, enlarged sample	Metabolic syndrome	29,998	8,306	0.88 (0.85–0.91)	<0.001
Without energy intake, enlarged sample	Depressive symptoms	29,998	1,525	0.90 (0.85–0.96)	0.002

Odds ratios correspond to a 1-SD increase in log-transformed PASTBI. The enlarged sample analysis omitted energy intake as a covariate, retained participants who did not complete the nutrition survey, and used the health interview and examination weight. CI, confidence interval; OR, odds ratio; PASTBI, physical activity–sitting time balance index; SD, standard deviation. PHQ-9, Patient Health Questionnaire-9.

Table S2. Test of the ratio constraint according to the handling of zero physical activity

Specification	MetS: PA OR	MetS: ST OR	MetS: P	Dep: PA OR	Dep: ST OR	Dep: P
log (physical activity+1)	0.97	1.12	0.009	0.98	1.26	<0.001
log (physical activity+10)	0.95	1.11	0.063	0.97	1.26	<0.001
log (physical activity+100)	0.92	1.10	0.711	0.97	1.27	0.001
log (physical activity), participants with any physical activity	0.91	1.10	0.972	1.01	1.20	0.011

Odds ratios correspond to a doubling of each exposure. P values test the ratio constraint. Dep, depressive symptoms; MetS, metabolic syndrome; PA, physical activity; ST, sitting time.

Table S3. Restricted cubic spline models with different numbers of knots

No. of knots	MetS: ΔAIC	MetS: P overall	MetS: P non-linearity	Dep: ΔAIC	Dep: P overall	Dep: P non-linearity
3	4.8	<0.001	<0.001	0.9	<0.001	0.014
4	1.7	<0.001	<0.001	0.1	<0.001	0.020
5	0.0	<0.001	<0.001	0.0	<0.001	0.018

ΔAIC denotes the difference from the model with the lowest Akaike information criterion within each outcome. Dep, depressive symptoms; MetS, metabolic syndrome.

Table S4. Associations of the physical activity–sitting time balance index with metabolic syndrome and depressive symptoms according to sex and age group

Outcome and subgroup	No. of participants	No. of cases	OR (95% CI)	P	P for interaction
**Metabolic syndrome**					
Sex					0.372
Men	11,385	3,773	0.85 (0.81–0.90)	<0.001	
Women	15,331	3,529	0.90 (0.86–0.95)	<0.001	
Age group					0.602
19–39 years	7,232	902	0.85 (0.77–0.94)	0.002	
40–64 years	12,487	3,572	0.89 (0.85–0.94)	<0.001	
65–74 years	4,536	1,779	0.85 (0.79–0.92)	<0.001	
≥75 years	2,461	1,049	0.93 (0.83–1.04)	0.196	
**Depressive symptoms**					
Sex					0.770
Men	11,385	392	0.87 (0.77–0.98)	0.021	
Women	15,331	940	0.91 (0.84–0.99)	0.032	
Age group					0.450
19–39 years	7,232	450	0.92 (0.80–1.06)	0.253	
40–64 years	12,487	526	0.92 (0.83–1.01)	0.086	
65–74 years	4,536	214	0.82 (0.70–0.96)	0.012	
≥75 years	2,461	142	0.86 (0.72–1.03)	0.099	

Odds ratios correspond to a 1-SD increase in log-transformed PASTBI. CI, confidence interval; OR, odds ratio; PASTBI, physical activity–sitting time balance index; SD, standard deviation.

Table S5. Distribution of the number of metabolic risk factors according to quartiles of the physical activity–sitting time balance index

No. of risk factors	Q1 (≤33)	Q2 (33–99)	Q3 (99–238)	Q4 (>238)	P
0–1 risk factor	3,217 (48.4)	3,330 (53.3)	3,522 (57.5)	3,652 (57.1)	<0.001
2 risk factors	1,527 (20.9)	1,351 (19.9)	1,358 (19.9)	1,457 (21.1)	0.330
≥3 risk factors	2,306 (30.6)	1,902 (26.9)	1,528 (22.6)	1,566 (21.8)	<0.001

Values are unweighted n (weighted %).

Table S6. Preferred Reporting Items for Complex Sample Survey Analysis (PRICSSA) checklist

Item	PRICSSA item	Reporting in this study	Location
1.1	Data collection dates	Six survey cycles were conducted from January to December in 2014, 2016, 2018, 2020, 2022, and 2024.	Methods, Study population
1.2	Data collection mode(s)	Health interviews and health behavior questionnaires and physical examinations at mobile examination centers, and face-to-face nutrition surveys in participants' homes.	Methods, Study population
1.3	Target population	Non-institutionalized Korean residents. The present analysis included adults aged 19 years or older.	Methods, Study population
1.4	Sample design	Stratified, multistage, clustered probability sampling with survey districts as primary sampling units.	Methods, Study population
1.5	Survey response rate(s)	Cumulative participation rates of 78.3%, 76.6%, 74.0%, and 69.4% for the 2013–2015, 2016–2018, 2019–2021, and 2022–2024 survey cycles.	Methods, Study population
2.1	Missingness rates	Missing PHQ-9 (n = 4,041), physical activity or sitting time (n = 359), covariates (n = 3,991), and metabolic syndrome components (n = 1,228). Complete case analysis was performed.	Figure 1, Table S7
2.2	Observation deletion	No observations were deleted from the survey design. Analyses were restricted to the analytic sample using domain (subpopulation) estimation.	Methods, Statistical analysis
2.3	Sample sizes	Unweighted numbers of participants and cases are reported for all estimates.	Tables 1–5
2.4	Confidence intervals and standard errors	Standard errors are reported for weighted means and 95% confidence intervals for all odds ratios.	Tables 1–5
2.5	Weighting	All analyses were weighted using the combined health interview, examination, and nutrition survey weight (wt_tot) divided by six for the pooled survey years. Unweighted models were fitted as a sensitivity analysis.	Methods, Statistical analysis; Table S1
2.6	Variance estimation	Taylor series linearization using variance estimation strata (kstrata, made unique within survey year; 160 strata) and primary sampling units (psu; 1,140 PSUs). Design degrees of freedom = 980.	Methods, Statistical analysis
2.7	Subpopulation analysis	Subgroup, stratified, and analytic-sample estimates were obtained by domain estimation within the full survey design.	Methods, Statistical analysis
2.8	Suppression rules	Not applied. All reported cells included at least 10 unweight cases.	Not applicable
2.9	Software and code	R version 4.3.3 with the survey and rms packages. Analysis code is available from the corresponding author on reasonable request.	Methods, Statistical analysis; Declarations
2.1	Singleton problem	No stratum contained a single primary sampling unit.	Methods, Statistical analysis
2.11	Public or restricted data	Public-use data files.	Declarations, Availability of data and materials
2.12	Embedded experiments	Not applicable.	Not applicable

Table S7. Characteristics of included and excluded participants

Characteristic	Included (n = 26,716)	Excluded (n = 9,619)	P
Age, years	51.6 ± 16.7	53.9 ± 18.1	<0.001
Women	15,331 (57.4)	5,163 (53.7)	<0.001
Elementary school or lower	4,828 (18.1)	1,590 (25.0)	<0.001
College or higher	10,269 (38.4)	2,120 (33.4)	<0.001
Lowest household income quartile	4,717 (17.7)	2,386 (25.2)	<0.001
Married or cohabiting	21,951 (82.2)	7,928 (82.4)	0.571
Current smoker	4,307 (16.1)	1,523 (20.0)	<0.001
Body mass index, kg/m^2	24.0 ± 3.6	24.0 ± 3.7	0.876
Sitting time, h/day	8.3 ± 3.5	8.5 ± 3.7	<0.001
Physical activity, MET-min/week, median (IQR)	792 (278–1,668)	612 (142–1,432)	<0.001
Depressive symptoms	1,332 (5.0)	391 (7.0)	<0.001
Metabolic syndrome	7,302 (27.3)	2,085 (32.9)	<0.001

Values are unweighted mean ± SD, median (IQR), or n (%). Percentages were calculated among participants with available data. IQR, interquartile range; MET, metabolic equivalent of task; SD, standard deviation.

Table S8. Sensitivity analysis for the survey period and the definition of physical activity

Analysis	Outcome	No. of participants	PASTBI per 1-SD, OR (95% CI)	P	Active participants, n	PA per doubling, OR	ST per doubling, OR	P for ratio constraint	Inactive participants, n	ST per doubling among inactive, OR (95% CI)
Excluding the 2020 and 2022 survey years	Metabolic syndrome	18,563	0.89 (0.85–0.94)	<0.001	16,138	0.91	1.09	0.890	2,425	1.04 (0.88–1.24)
	Depressive symptoms	18,563	0.91 (0.84–0.99)	0.037	16,138	1.04	1.26	0.002	2,425	1.58 (1.16–2.14)
Standard GPAQ scoring of physical activity	Metabolic syndrome	26,679	0.88 (0.84–0.91)	<0.001	18,797	0.89	1.06	0.142	7,882	1.15 (1.04–1.28)
	Depressive symptoms	26,679	0.93 (0.87–1.01)	0.070	18,797	1.10	1.22	<0.001	7,882	1.53 (1.22–1.91)
Physical activity excluding the walking item	Metabolic syndrome	26,716	0.84 (0.81–0.88)	<0.001	10,073	0.90	1.07	0.547	16,643	1.12 (1.04–1.19)
	Depressive symptoms	26,716	1.05 (0.97–1.12)	0.228	10,073	1.08	1.10	0.118	16,643	1.45 (1.24–1.68)

Standard GPAQ scoring combined vigorous- and moderate-intensity activity in the work and leisure domains with walking or cycling for transport, without the separate walking item. Odds ratios for physical activity and sitting time correspond to a doubling of each exposure among participants with any physical activity. Inactive participants are those with no physical activity under each definition, for whom PASTBI equals zero. CI, confidence interval; GPAQ, Global Physical Activity Questionnaire; OR, odds ratio; PA, physical activity; PASTBI, physical activity–sitting time balance index; SD, standard deviation; ST, sitting time.
